# Catalytically inactive PARP1 protein drives PARP inhibitor induced hematological toxicity

**DOI:** 10.64898/2026.09.21.753312

**Published:** 2026-09-25

**Authors:** Xiaohui Lin, Zhengping Shao, Denitsa Yaneva, Wenxia Jiang, Demis Menolfi, Seema Khattri Bhandari, Brian J Lee, Mirjam Schmucker, Faye Yan, Alan E Tomkinson, Julian Stingele, Shan Zha

## Abstract

Dual PARP1/2 inhibitors (PARPi) selectively eliminate BRCA1/2-deficient cancers and represent the first targeted therapy for homologous recombination (HR)-deficient cancers. However, their use in maintenance therapy is limited by severe anemia and an increased risk for therapy-related leukemia. These toxicities are unexpected because PARP1 loss, which eliminates most DNA-damage-induced PARylation, does not cause anemia in mice. In contrast, PARP2 loss or catalytic inactivation causes anemia, motivating the development of PARP1-selective inhibitors. Using wild-type (WT), Parp1-/- and Parp2-/- mice, we show that hematopoietic toxicity of FDA-approved PARPi is driven primarily by inactive PARP1 rather than PARP2 inhibition. Accordingly, PARP1-selective inhibitors also cause PARP1-dependent anemia. Somatic expression of catalytically inactive Parp1 (Parp1E988A) causes lethal bone marrow failure, not found with somatic deletion of both Parp1&2. Mechanistically, inactive PARP1 obstructs the repair of diverse DNA lesions, including gaps, nicks, and Top1-cc, in contrast to the nick-selectivity of Parp2. In cells, inactive PARP1 compromises PARP2 recruitment to DNA lesions and causes severe genomic instability and mitotic bridges absent in Parp1&2-null cells. Thus, PARPi-induced hematopoietic toxicity is driven primarily by PARP1 inactivation, informing the design and use of next-generation PARP inhibitors.

## Full Text Availability

The license terms selected by the author(s) for this preprint version do not permit archiving in PMC. The full text is available from the preprint server.

